# Proteomic Insight into the Response of Arabidopsis Chloroplasts to Darkness

**DOI:** 10.1371/journal.pone.0154235

**Published:** 2016-05-03

**Authors:** Jing Wang, Qingbo Yu, Haibo Xiong, Jun Wang, Sixue Chen, Zhongnan Yang, Shaojun Dai

**Affiliations:** 1 Department of Mathematics, College of Mathematics and Science, Shanghai Normal University, Shanghai, P.R. China; 2 Institute of Plant Gene Function, Shanghai Normal University, Shanghai, P.R. China; 3 Department of Biology, College of Life and Environmental Sciences, Shanghai Normal University, Shanghai, P.R. China; 4 Department of Biology, Genetics Institute, Plant Molecular and Cellular Program, Interdisciplinary Center for Biotechnology Research, University of Florida, Gainesville, United States of America; University of California—Davis, UNITED STATES

## Abstract

Chloroplast function in photosynthesis is essential for plant growth and development. It is well-known that chloroplasts respond to various light conditions. However, it remains poorly understood about how chloroplasts respond to darkness. In this study, we found 81 darkness-responsive proteins in Arabidopsis chloroplasts under 8 h darkness treatment. Most of the proteins are nucleus-encoded, indicating that chloroplast darkness response is closely regulated by the nucleus. Among them, 17 ribosome proteins were obviously reduced after darkness treatment. The protein expressional patterns and physiological changes revealed the mechanisms in chloroplasts in response to darkness, e.g., (1) inhibition of photosystem II resulted in preferential cyclic electron flow around PSI; (2) promotion of starch degradation; (3) inhibition of chloroplastic translation; and (4) regulation by redox and jasmonate signaling. The results have improved our understanding of molecular regulatory mechanisms in chloroplasts under darkness.

## Introduction

Chloroplasts perform important functions essential for plant growth and development, such as light-harvesting, photosynthetic electron transport, Calvin cycle, and biosynthesis of fatty acids, lipids, amino acids, nucleotides, hormones, alkaloids, and isoprenoids [1]. Chloroplasts are sensitive to the changes of light quantity and quality. Under high illumination conditions, chloroplasts are damaged due to the absorption of light exceeds the capacity of the photosynthetic apparatus to dissipate the excess energy [2]. At extremely low light intensities, the chloroplasts of Arabidopsis plants showed a changed number of grana stacks and a decreased chlorophyll and protein content [3]. However, chloroplasts have evolved numerous strategies to improve the efficiency of photosynthesis and protect against the damaging effects of abnormal light conditions, e.g., induction of gene reprogram, adjustment in reaction center stoichiometry, changes of antenna size and ribulose bisphosphate carboxylase oxygenase (RuBisCO) level. In addition, antioxidants, antioxidant enzymes, and chaperones were changed in response to various light conditions [4].

Acclimation strategies must concomitantly meet the challenges of light alteration to darkness [5]. On a daily basis, photosynthesis results in carbon fixation and starch accumulation, then degradation of starch supports metabolic and growth activities at night. Until the end of the night, only about 10% of the starch is left [6]. The photosynthetic carbon assimilation is strictly cycled as light/dark periods [7]. In the light, the linear electron flow (LEF) pathway together with cyclic electron flow (CEF) pathway results in the formation of ATP and photosynthetic NADPH, which can be used to drive the reactions of the Calvin cycle for CO_2_ assimilation in stroma [8]. Reducing equivalents (NADPH) from the photosynthetic electron flow is transferred to thiol enzymes containing redox-active cysteine residues, allowing modulation of their enzymatic activities [9]. Thus, thioredoxin (Trx)-regulated Calvin cycle enzymes are light dependent, which are activated in the light and inactivated in the dark [10]. In the dark, carbohydrates are broken down to produce ATP and reducing power (non-photosynthetic NADPH), which attributed to remain electron flow through chlororespiration [11]. As a component of the photosynthetic machinery, chlorophyll a cannot be produced in the dark [12]. The chloroplastic ATP-ADP ratio is very low in the dark, but increases in the light [13]. Therefore, the process of protein synthesis in the chloroplast as the extremely energy demanding process is sequestered in the dark but stable in the light [14]. However, when Arabidopsis growing in a regular light/dark cycle, the diurnal gene expression is regulated not only by light but also by sugar and clock [15]. Several photosynthetic genes are showed with circadian clock-regulated mode [16]. The first observations of clock-regulated gene expression came from studies where the induction of the gene expression from chlorophyll a/b binding proteins (CAB), the small subunit of RuBisCO, and PsaD as the photosystem I core protein was clearly seen in the predawn [17]. The working period of the photosynthetic machinery and the time of starch turnover are clock-controlled processes [18–19]. In addition, rhythmic endogenous sugar signals can influence circadian rhythms in Arabidopsis [20]. The addition of sucrose to the growth medium can sustain circadian rhythms when they are grown in continuous dark [21].

Only 120–130 proteins were encoded in chloroplasts, most of which are components of the organelle’s gene expression machinery and its photosynthetic apparatus, and are organized in nucleoids. About 4000 proteins in the chloroplasts are nucleus-encoded [22]. However, about 2000 non-redundant proteins have been identified in the chloroplast so far, belonging to three compartments: stroma (∼344 proteins), thylakoids (∼400), and envelope membranes (∼400) [23–24], and the others are ribosome proteins and nucleoid proteins.

Large-scale differential display techniques have been employed to study the chloroplast protein composition in response to changing environmental conditions, which provide information about the function of proteins that are altered, and reflect the more realistic changes of chloroplasts. With the maturation of proteomic workflows, quantitative information for plastid proteins became available. These new technologies, in combination with the availability of sequenced plant genomes, allow for system-level analysis of chloroplast biology, including chloroplast development, signaling, and interaction networks [25–27]. In the present study, the changes of chloroplast proteome in Arabidopsis in response to darkness were analyzed using tandem mass tag (TMT)-based proteomics. The differentially expressed proteins provide important insight into the darkness response of Arabidopsis chloroplasts.

## Materials and Methods

### Plant growth and Chloroplast preparation

**Fig 1:**
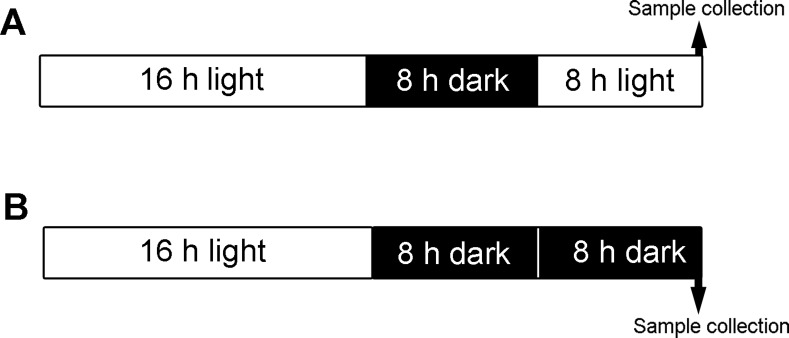
Treatment and sampling scheme of *Arabidopsis thaliana* leaves at seven-leaf rosette stage. (A) Control plants were grown under normal cycle of 16 h light/8 h dark; (B) Treated plants were grown under 8 h extension of dark after normal cycle.

*Arabidopsis thaliana* (Columbia-0) seeds were vernalized under 4°C condition for 2 days, and then grown for 3 weeks at 21°C in a 16 h light (120 μmol photons m^–2^ s^–1^)/8 h dark cycle in soil. The seedlings of control samples were under normal light/dark cycle, but the treatment samples were kept in the dark for additional 8 h (Fig 1). For dark treated plants and control plants, fully expanded leaves from three-week-old plants were used for intact chloroplast preparation as previously described [28]. The slurry of leaves were filtered through six layers of miracloth, 100μm cell strainer (Biologix, USA), 40μm cell strainer (Biologix, USA), and then pelleted by centrifugation. Chloroplasts were collected and stored in -80°C.

### Chlorophyll fluorescence, P700 Parameters, and Chlorophyll content analysis

In vivo Chlorophyll (Chl) a fluorescence of whole plants was recorded using an imaging Chl fluorometer (Imaging PAM; Walz, Germany). Both dark treated plants and control plants were put into darkness for 15min, then dark-treated plants were exposed to a pulsed, blue measuring beam (1 Hz, intensity 4; F_0_), and a saturating light flash (intensity 4).

The chlorophyll fluorescence, and P700 redox state were measured using a DUAL-PAM-100 instrument (Heinz Walz, Effeltrich, Germany) according to the method of Yamori et al. [29] with minor modifications. The effective quantum yield of PSII is termed as Y(II), and the quantum yield of PSI [Y(I)] is defined by the proportion of P700 in reduced state. Y(CEF)/Y(II) was used to estimate the extent of CEF as described in a previous study [30], which was calculated as: Y(CEF)/Y(II) = [Y (I)—Y(II)]/Y(II).

For the measurement of chlorophyll *a* and chlorophyll *b* contents, approximately 20 rosette leaves from three plants were pooled, and chlorophylls were extracted from pooled sample using an acetone method [31]. Extracts were centrifuged at 15,000g for 10 min at 4°C. The supernatant was diluted with water to a final acetone concentration of 80%. The chlorophyll *a* and chlorophyll *b* contents were determined at 645 nm and 663 nm using a spectrophotometer [31].

### Determination of Soluble Sugar and Starch content

Soluble sugar was extracted from leaf discs sampled three times. The leaf discs were incubated in 80% (v/v) ethanol for 30 min at 80°C and then washed 4 times with ethanol 80% (v/v). Soluble Sugar was measured from the extracts in ethanol using an Anthrone reagent -based protocol [32]. Starch was determined using Iodine Colorimety as previously described [33]. Starch and total soluble sugar content were determined as the percentage of sugar (μg) in fresh leaves (g).

### Determination of enzyme activities

The activity of RuBisCO and Triose-phosphate isomerase (TPI) were determined by Plant RuBisCO ELISA Kit and Plant TPI ELISA Kit (R&D Systems China Co., Ltd.). According to the instruction of the ELISA Kit, using purified RuBisCO (TPI) to coat microtiter plate wells, and making solid-phase antibody, then add samples to wells, and combine with RuBisCO (TPI) antibody with HRP labeled, forming antibody-antigen-enzyme-antibody complex. After washing completely, TMB substrate solution was added, and the TMB substrate becomes blue color. The reaction is terminated by the addition of a sulphuric acid solution and the color change is measured spectrophotometrically at a wavelength of 450 nm. The concentrations of RuBisCO (TPI) in the samples were then determined by comparing the O.D. of the samples to the standard curve and calculated as the activity of enzyme (U) per fresh leaves extract (ml). The activity of each enzyme was represented by the average of three biological replicates.

### Western blotting

The proteins from leaves for Western blotting were extracted with an extraction buffer (25 mM Tris–HCl, pH 8.6, 50 mM NaCl, 6 mM NH_4_Cl, 10 mM MgCl_2_•6H_2_O, and 5% (v/v) Triton X-100). Protein concentration were quantified using a 2-D Quant kit (GE Healthcare, USA). The proteins were separated on 12% SDS-PAGE, followed by transferring onto a BioTrace polyvinylidene difluoride membrane (Bio-Rad Laboratories). The membrane for leaf proteins was immunoreacted with the RbcL, Lhcb1, and Organelle Detection Western Blot Cocktail antibodies (Abcam, Cambridge, UK), and the membrane for chloroplast proteins was immunoreacted with the Organelle Detection Western Blot Cocktail antibodies, the Lhcb1, PSAA, RbcL, and D1 antibodies. The signals were developed using the enhanced chemiluminescence, and the signal intensity was quantified using Quantity One software (Bio-Rad Laboratories, Inc., USA).

### Protein extraction

Chloroplast extracts were pooled from three biological replicates and dissolved in 450 μl HM buffer (10mM HEPES-KOH pH 7.5, 1M MgCl_2_). After incubation on ice for 10 min, chloroplast lysate was incubated in a buffer containing 4% CHAPS, 7M Urea, 2M Thiourea, Tris-HCl, pH 8.0–9.0, 5 mM MgCl_2_, 20% Protease Inhibitor Cocktail (Sigma, USA), followed by sonication for 15s, three times, and then centrifuged at 1,400g for 10 min at 4°C. Protein concentration in the supernatant was determined using a 2-D Quant kit (GE Healthcare, USA) according to the manufacturer’s instructions.

### Quantitative proteomics using LC-MS/MS

*Protein Digestion*, *TMT Labeling and Strong Cation Exchange Fraction—*The clarified protein lysates was reduced, alkylated, and trypsin digested before subjected to labeling with TMT reagent (Thermo Scientific™, Rockford, USA) as previously described [34–35]. The peptide was desalted by Strata X C18 SPE column (Phenomenex) and vacuum-dried. Peptide was reconstituted in 0.5 M TEAB and processed according to the manufacturer’s protocol for 6-plex TMT kit. Control samples were labelled with the tags TMT-126, TMT-127 and TMT-128, while dark-treated samples were labelled with the tags TMT-129, TMT-130 and TMT-131. Briefly, one unit of TMT reagent (defined as the amount of reagent required to label 100 μg of protein) was thawed and reconstituted in 24 μl acetonitrile (Fisher Chemical, USA). The peptide mixtures were then incubated for 2 h at room temperature and pooled, desalted and dried by vacuum centrifugation. After labeling, two samples (control and dark-treated) were combined and lyophilized. The peptide was then fractionated by high pH reverse-phase HPLC using Agilent 300Extend C18 column (5 μm particles, 4.6 mm ID, 250 mm length). The peptides were first separated with a gradient of 2% to 60% acetonitrile in 10 mM ammonium bicarbonate pH 8 over 80 min into 80 fractions. The peptides were combined into 18 fractions and dried by vacuum centrifuging.

*Reverse Phase Nanoflow HPLC and Tandem Mass Spectrometry*—Peptides were dissolved in 0.1% FA, directly loaded onto a reversed-phase pre-column (Acclaim PepMap 100, Thermo Scientific). Peptide separation was performed using a reversed-phase analytical column (Acclaim PepMap RSLC, Thermo Scientific). The gradient was comprised of an increase from 6% to 25% solvent (0.1% FA in 98% acetonitrile) over 28 min, 25% to 35% in 6 min and climbing to 80% in 2 min then holding at 80% for the last 4 min, all at a constant flow rate of 300 nl/min on an EASY-nLC 1000 (UHPLC system). The resulting peptides were analyzed by Q Exactive^TM^ Plus hybrid quadrupole-Orbitrap mass spectrometer (ThermoFisher Scientific).

Intact peptides were detected in the Orbitrap at a resolution of 70,000. Peptides were selected for MS/MS using NCE setting as 30; ion fragments were detected in the Orbitrap at a resolution of 17,500. A data-dependent procedure that alternated between one MS scan followed by 20 MS/MS scans was applied for the top 20 precursor ions above a threshold ion count of 2E^4^ in the MS survey scan with 10 s dynamic exclusion. The electrospray voltage applied was 2.0 kV. Automatic gain control (AGC) was used to prevent overfilling of the ion trap; 5 E^4^ ions were accumulated for generation of MS/MS spectra. For MS scans, the m/z scan range was 350 to 1600 Da. Fixed first mass was set as 100 m/z.

### Protein Identification and Quantification

The resulting MS/MS data were processed using Mascot search engine (v.2.3.0). Tandem mass spectra were searched against Uniprot_Arabidopsis database concatenated with reverse decoy database. The searching criteria include: trypsin/P was specified as cleavage enzyme allowing up to 2 missing cleavages, mass error was set to 10 ppm for precursor ions and 0.02 Da for fragment ions. Carbamidomethyl on Cys, TMT-6 plex (N-term) and TMT-6 plex (K) were specified as fixed modification and oxidation on Met was specified as variable modifications. FDR was adjusted to less than 1% and peptide ion score was set more than 20.

For quantitative changes, the protein ratio dark-treated/control was computed according to the tagging design, i.e. TMT129+130+131/TMT126+127+128, ratios with p-values less than 0.05 present in all three technical replicates were considered significant. Only the significant ratios from all three replicates were used to calculate the average ratio for the protein. It should be noted that each p-value was generated based on quantitative information derived from at least three independent peptides in each replicate. The protein was determined as differentially expressed with a threshold of fold change (cutoff of 1.3 for increased expression and 0.77 for decreased expression).

Protein annotations were obtained from the UniProt-GOA database (http://www.ebi.ac.uk/GOA/), together with the InterProScan software analysis based on protein sequence alignment method. The gene numbers were derived from *Arabidopsis* TAIR database (http://www.arabidopsis.org/). The localizations of the proteins were determined through Swissprot database, AT-CHLORO database and PPDB database. The proteins were classified according to UniProt-GOA database, InterProScan software, and KEGG database.

The mass spectrometry proteomics data have been deposited to the ProteomeXchange Consortium via the PRIDE [36] partner repository with the dataset identifier PXD003516.

## Results

### Effect of darkness on Chl fluorescence, P700 Parameters, and Chlorophyll content

**Fig 2:**
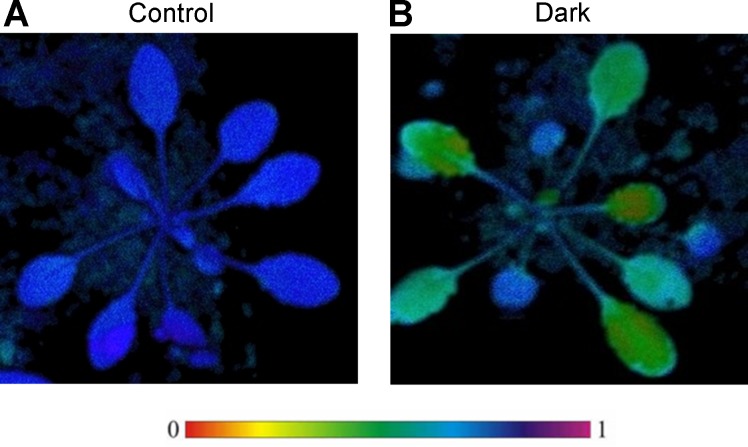
Photosynthetic performance of *Arabidopsis thaliana* plants. (A) Control plants; (B) Dark-treated plants. The photosynthetic parameters F_V_/F_M_ were measured at the seven-leaf rosettes stage.

The Arabidopsis plants were subjected to Chl *a* fluorescence measurements to monitor photosynthetic performance of PSII. The maximum quantum yield of PSII (F_V_/F_M_) was reduced in seedlings under dark treatment (Fig 2). This indicated that the efficiency of PSII was decreased in seedlings in the dark compared to seedlings in the light. In addition, the Y(CEF)/Y(II) in the dark was about 3.77, while Y(CEF)/Y(II) in the light was only about 1.07. This reflected that CEF was enhanced under darkness (Table 1).

**Table 1:** Photosynthetic parameters and pigment contents in leaves.

	Photosynthetic parameter	Leaf pigment content (mg/g fresh leaf)			
	Y(CEF)/Y(II)	Chl *a*	Chl *b*	Chl *a+b*	Chl *a/b*
Control	1.075±0.005	0.683±0.002	0.406±0.019	1.089±0.020	1.684
Dark-treated	3.760±0.012*	0.600±0.003*	0.487±0.018*	1.087±0.017	1.338*

The photosynthetic parameter and leaf pigments were quantified. Mean values ± SD are shown. Chl a, chlorophyll a; Chl b, chlorophyll b.

* Value is significantly different from the respective control leaves (t-test value p<0.05).

To quantify the difference of leaf coloration between control and dark-treated plants, chlolophyll contents in leaves were analyzed (Table 1). The total Chl content was unchanged in dark-treated leaves. However, the Chl *a*/*b* ratio was 1.338 in dark-treated samples, which was lower than that in the light, due to the increase of Chl *b* and decrease of Chl *a* under dark treatment (Table 1).

### Effect of darkness on starch and sugar content in leaves

**Fig 3:**
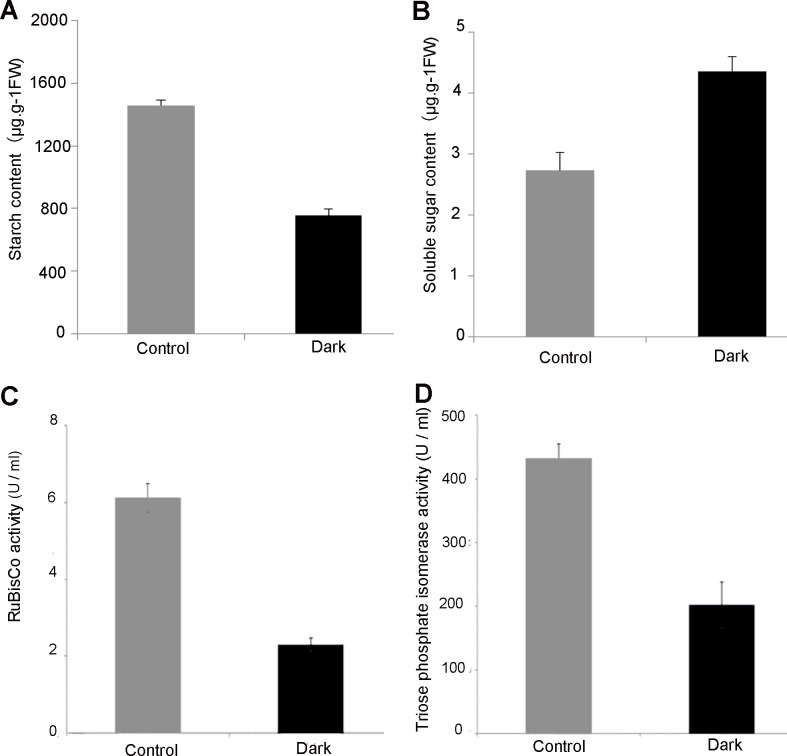
Starch and soluble sugar contents and activities of RuBisCO and triose phosphate isomerase in *Arabidopsis thaliana* leaves. (A) Starch content in leaves; (B) Soluble sugar content in leaves; (C) RuBisCO activity; (D) Triose-phosphate isomerase activity.

The starch content in dark-treated leaves was obviously reduced than that in light (Fig 3A), but the soluble sugar content in leaves under dark treatment was 1.6-fold higher than that in light (Fig 3B). It is well known that the carbohydrates are altered between carbon accumulation during the day and carbon consumption at night. The night (darkness) was supposed to enhance starch degradation [37–38]. Thus, our results implied that the increased soluble sugars were mainly generated from starch degradation in leaves under darkness.

### Effect of darkness on RuBisCO and TPI activity

RuBisCO is involved in the first step of carbon fixation, and functions as the primary rate-limiting enzyme in Calvin cycle. After darkness treatment, the RuBisCO activity was reduced nearly two-fold than that under normal condition (Fig 3C). In addition, TPI is involved in the regeneration of ribulose 1,5-bisphosphate (RuBP) as the substrate of RuBisCO. In our results, TPI activity was also reduced under dark treatment (Fig 3D). All these results implied that the darkness inhibited enzyme activities in carbon fixation.

### Profiling the chloroplast proteome by quantitative mass spectrometry

**Fig 4:**
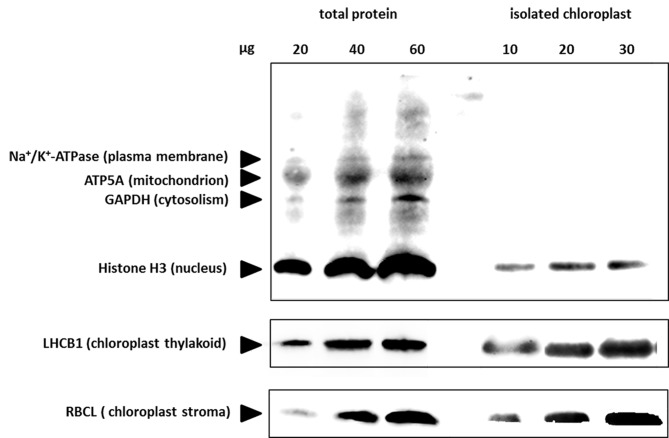
Immunological estimation of contamination in the isolated chloroplasts. Immunoblot analyses were performed on dilution series of total protein extract (left) and isolated chloroplasts (right). The indicated amounts of each protein were loaded, and the blots were probed with antibodies against proteins from various different subcellular compartments, as indicated. ATP5A, ATP synthase subunit α; GAPDH, glyceraldehyde-3-phosphate dehydrogenase; LHCB I, Light-harvesting chlorophyll protein complex II subunit B1; RBCL, large subunit of Rubisco.

To evaluate the purity of chloroplast sample, Western blotting was conducted by using antibodies against the marker proteins from plasma membrane (Na^+^/K^+^-ATPase), mitochondrion (ATP5a), cytoplasm (GAPDH), nucleus (histone H3), chloroplast thylakoid (LHCB1), and chloroplast stroma (RBCL), respectively. The results indicated that the chloroplast sample mainly has contaminant of nucleus (Fig 4).

Chloroplasts protein digests were analyzed on a hybrid Quadrupole-Orbitrap (QE) mass spectrometer using data dependent acquisition (DDA) and dynamic exclusion. The mass spectrometry data validation was shown in S1 Fig. The distribution of mass error of identified peptides was near to zero and most of them were less than 0.02 Da (S1 Fig). The mass accuracy of mass spectrometry data clearly meets the specifications. In addition, the lengths of most identified peptides were between eight and 16 amino acids (S1 Fig).

**Fig 5:**
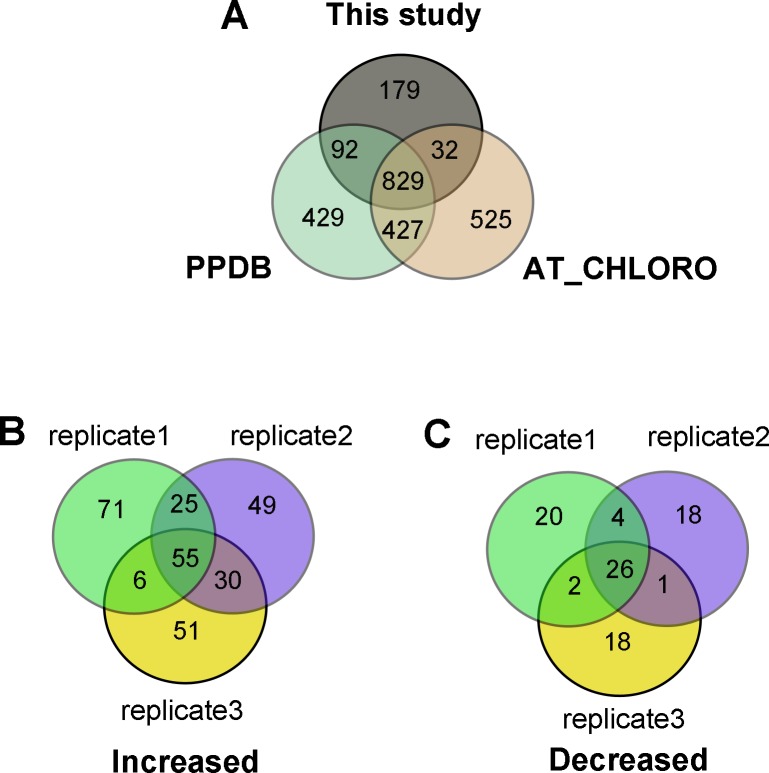
Number of chloroplastic proteins identified in this study. (A) The number of chloroplastic proteins identified in this study compared with PPDB database and AT_CHLORO database; (B) Darkness-increased chloroplastic proteins; (C) Darkness-decreased chloroplastic proteins.

In total, 1386 proteins were unambiguously identified using Mascot search engine (v.2.3.0) (S1 and S2 Tables). Among them, 953 proteins were annotated as chloroplast proteins in PPDB database (http://ppdb.tc.cornell.edu/) and a published AT_CHLORO database (http://at-chloro.prabi.fr/at_chloro/) (Fig 5A). Furthermore, 179 proteins with predicted chloroplast localization (http://www.arabidopsis.org/) were not present in available chloroplastic database (Fig 5A).

**Fig 6:**
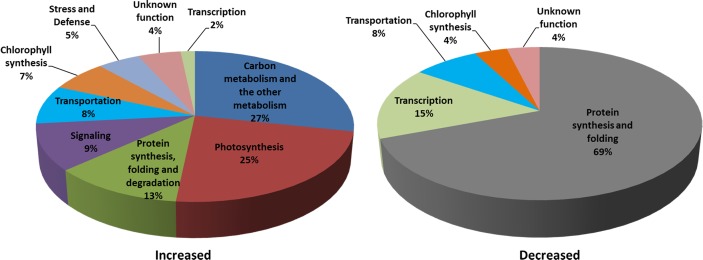
Pie chart representation of the functional groups of darkness-responsive proteins in chloroplasts. (A) Darkness-increased proteins; (B) Darkness-decreased proteins.

When comparing dark-treated sample with control sample, there are 81 darkness-responsive proteins. Among them, 55 proteins were increased and 26 proteins were decreased (Fig 5B and 5C; Tables 2 and 3). On the basis of the Gene Ontology and information from the literature, these darkness-responsive proteins were classified into nine functional categories, which were photosynthesis, carbohydrate metabolism, chlorophyll synthesis, protein synthesis, protein folding and turnover, transport, signaling, stress and defense, and function unknown (Fig 6). The metabolism-related proteins accounted for 27%, and photosynthesis proteins accounted for 25% of the darkness-increased proteins. In addition, components of protein synthesis apparatus accounted for 69% of the darkness-decreased proteins.

**Table 2:** Darkness-increased proteins in chloroplast.

No ^a^	Protein name ^b^	Protein Number ^c^	Gene Number ^d^	Sub-localization ^e^	D/L Ratio ± SD ^f^
	**Photosynthesis (15)**^g^				
1	Light-harvesting chlorophyll protein complex II subunit B1 (LHCB1.4)	Q39142	AT2G34430	T	1.59±0.06
2	Chlorophyll a-b binding protein (CP26)	Q9XF89	AT4G10340	T	1.43±0.04
3	Photosystem I light harvesting complex gene2 protein (LHCA2)	Q9M320	AT3G61470	T	1.45±0.22
4	Photosystem I P700 chlorophyll a apoprotein A1 (PSAA)	P56766	ATCG00350	T	1.65±0.13
5	Sulfite reductase [ferredoxin] (Fd)	Q9LZ66	AT5G04590	Tl	1.37±0.02
6	PGR5-like protein 1A (PGRL1A)	Q8H112	AT4G22890	T	1.33±0.09
7	PGR5-like protein 1B (PGRL1B)	Q8GYC7	AT4G11960	T	1.42±0.06
8	Serine/threonine-protein kinase (STN7)	Q9S71b	AT1G68830	T	1.32±0.03
9	Serine/threonine-protein kinase (STN8)	Q9LZV4	AT5G01920	T	1.43±0.10
10	PsbP	Q42029	AT1G06680	T	1.34±0.13
11	PsbQ	Q9XFT3	AT4G21280	T	1.44±0.09
12	PsbP-like protein (PPL1)	P82538	AT3G55330	Tl	1.50±0.03
13	PsbP domain-containing protein 5 (PPD5)	P82715	AT5G11450	Tl	1.34±0.04
14	Protein curvature thylakoid 1A (CURT1A)	O04616	AT4G01150	T	1.40±0.05
15	Rieske (2Fe-2S) domain-containing protein (PSB33)	Q9C9I7	AT1G71500	T	1.37±0.03
	**Chlorophyll and carotenoid synthesis (4)**				
16	Coproporphyrinogen-III oxidase 1 (CPOX)	Q9LR75	AT1G03475	S	1.45±0.08
17	Magnesium-protoporphyrin IX monomethyl ester [oxidative] cyclase (CRD1)	Q9M591	AT3G56940	Ts	1.40±0.10
18	Protochlorophyllide reductase like protein (POR C)	Q0WVW0	AT1G03630	Ts	1.53±0.09
19	Lycopene beta/epsilon cyclase protein (CrtL)	Q0WVA1	AT2G32640	T	1.61±0.12
	**Carbon metabolism and the other metabolism (15)**				
20	Ribulosebisphosphate carboxylase small chain (RBCS)	Q0WVH4	AT1G67090	S	1.53±0.07
21	Ribulosebisphosphate carboxylase large chain (RBCL)	O03042	ATCG00490	S	1.58±0.05
22	Sedoheptulose-1,7-bisphosphatase (SBPase)	P46283	AT3G55800	S	1.47±0.08
23	Transaldolase-like protein (TAL)	F4IC59	AT1G12230	S	1.57±0.04
24	Triosephosphateisomerase (TPI)	A8MRE8	AT2G21170	S	1.40±0.01
25	Fructose-bisphosphatealdolase(FBA)	F4IGL5	AT2G21330	S	1.60±0.08
26	Fructose-bisphosphatealdolase 3(FBA3)	Q9ZU52	AT2G01140	S	1.48±0.13
27	Phosphoenolpyruvate carboxylase family protein (PEPCK)	Q501F7	AT1G21440	S	1.37±0.05
28	Plastidial pyruvate kinase 1 (PKP1)	Q9LIK0	AT3G22960	S	1.47±0.09
29	Dihydrolipoyl dehydrogenase 1 (LPD1)	A8MS68	AT3G16950	S	1.34±0.06
30	Glutamine synthetase (GS)	B9DGD1	AT1G63770	S	1.64±0.06
31	Carbamoyl-phosphate synthase large chain (VEN3)	Q42601	AT1G29900	S	1.44±0.07
32	4-hydroxy-3-methylbut-2-enyl diphosphate synthase (HDS)	B3H725	AT5G60600	S	1.38±0.02
33	Ferredoxin—nitrite reductase ((NiR)	Q39161	AT2G15620	S	1.32±0.01
34	Amidophosphoribosyltransferase 2(ATase 2)	Q9STG9	AT4G34740	Chl	1.47±0.06
	**Transcription (1)**				
35	CCR-like (CCL)	Q96500	AT3G26740	Chl	1.63±0.06
	**Protein synthesis, folding, and degradation (7)**				
36	Ribosome-recycling factor (RRF)	Q9M1X0	AT3G63190	S	1.42±0.05
37	Peptidyl-prolylcis-trans isomerase (FKBP16-1)	Q944B0	AT4G26555	Tl	1.43±0.05
38	Peptidyl-prolylcis-trans isomerase (FKBP16-4)	Q9SR70	AT3G10060	Tl	1.33±0.03
39	Peptidyl-prolylcis-trans isomerase (FKBP19)	Q9LYR5	AT5G13410	Tl	1.49±0.13
40	Peptidyl-prolylcis-trans isomerase (CYP20-3)	F4IX26	AT3G62030	S	1.46±0.06
41	FtsH extracellular protease family protein (FtsH)	F4J3N2	AT3G04340	Chl	1.42±0.09
42	Protease DEG 8 (DEG8)	F4KFV6	AT5G39830	Tl	1.32±0.02
	**Transportation (4)**				
43	Outer envelope protein 64 (Toc64)	Q9LVH5	AT3G17970	OE	1.38±0.09
44	Preprotein translocase subunit (SCY1)	Q38885	AT2G18710	T	1.59±0.09
45	Inorganic phosphate transporter 2-1(PHT2;1)	Q38954	AT3G26570	IE	2.48±0.06
46	K^+^ efflux antiporter 3 (KEA3)	Q9M0Z3	AT4G04850	T	1.44±0.06
	**Signaling (5)**				
47	Rhodanese/Cell cycle control phosphatase superfamily protein 9 (Str9)	O48529	AT2G42220	S	1.48±0.08
48	Rhodanese/cell cycle control phosphatase superfamily protein	F4J9G2	AT3G59780	T	1.49±0.02
49	Rhodanese-like domain-containing protein 4 (TROL)	Q9M158	AT4G01050	T	1.39±0.03
50	Allene oxide synthase (AOS)	Q96242	AT5G42650	T	1.41±0.02
51	Allene oxide cyclase 4 (AOC4)	Q93ZC5	AT1G13280	S	1.47±0.07
	**Stress and Defense (2)**				
52	Acclimation of photosynthesis to environment protein (APE1)	Q2HIR7	AT5G38660	T	1.52±0.10
53	Resistance to phytophthora 1 protein (RPH1)	F4IN59	AT2G48070	Chl	1.64±0.04
	**Unknown (2)**				
54	Uncharacterized protein	F4IYD5	AT3G01060	Chl	1.48±0.15
55	Uncharacterized protein	Q9SGU7	AT1G64680	Chl	2.25±0.21

^a^ Numberical list of dark-responsive proteins.

^b^ Protein name and the abbreviation commonly used for the protein. The proteins were classified according to their functions.

^c^ Protein number given by Uniprot_Arabidopsis database.

^d^ Gene number, by converting identified protein ID to gene number in TAIR database.

^e^ Subcellular localization of each chloroplastic protein according to PPDB database.

^f^ D/L Ratio ± SD is shown as protein ratios with standard deviations.

^g^ Functional group with number of proteins in this group. The abbreviations: D, dark-treated samples; L, control samples. SD, standard deviation. D/L means protein abundance value of dark sample divided by value of control sample. SD: standard deviation. Chl: chloroplast; IE: envelope-inner-integral; S: plastid stroma; R: plastid ribosome; N: plastid nucleoid.

**Table 3:** Darkness-decreased proteins in chloroplast.

No ^a^	Protein name ^b^	Protein Number ^c^	Gene Number ^d^	Sub-localization ^e^	D/L Ratio ± SD ^f^
	**Chlorophyll synthesis (1)**^g^				
1	Magnesium-chelatase subunit ChlD	Q9SJE1	AT1G08520	Chl	0.72±0.04
	**Transcription (4)**				
2	chloroplast RNA-binding protein 33 (PDE322)	Q39061	AT3G52380	S	0.67±0.03
3	31-kDa RNA binding protein (RBP31)	Q94EH5	AT4G24770	S	0.53±0.01
4	RNA-binding (RRM/RBD/RNP motifs) family protein(PSRP2)	Q8VYM4	AT3G52150	R	0.56±0.02
5	plastid transcriptionally active 5(pTAC5)	A1A6M1	AT4G13670	N	0.71±0.03
	**Protein synthesis (18)**				
6	Translation initiation factor IF-2 (FUG1)	Q9SHI1	AT1G17220	S	0.65±0.03
7	Putative ribosomal protein S1 (RPS1)	Q93VC7	AT5G30510	R	0.59±0.01
8	30S ribosomal protein S3 (RPS3)	P56798	ATCG00800	R	0.72±0.01
9	30S ribosomal protein S5 (RPS5)	P93014	AT2G33800	R	0.35±0.00
10	30S ribosomal protein S20 (RPS20)	Q9ASV6	AT3G15190	R	0.44±0.02
11	50S ribosomal protein L1(RPL1)	Q9LY66	AT3G63490	R	0.50±0.04
12	50S ribosomal protein L2 (RPL2)	P56791	ATCG00830	R	0.70±0.03
13	Ribosomal protein L4 (RPL4)	Q0WW46	AT1G07320	R	0.44±0.03
14	Ribosomal L5P family protein (RPL5P)	O04603	AT4G01310	R	0.36±0.04
15	50S ribosomal protein L9 (RPL9)	P25864	AT3G44890	R	0.45±0.02
16	Ribosomal protein L10 family protein (RPL10)	B5X0P0	AT5G13510	R	0.53±0.02
17	50S ribosomal protein L12-1 (RPL12)	P36210	AT3G27830	R	0.55±0.02
18	50S ribosomal protein L14 (RPL14)	P56792	ATCG00780	R	0.67±0.02
19	50S ribosomal protein L15 (RPL15)	P25873	AT3G25920	R	0.53±0.03
20	50S ribosomal protein L21 (RPL21)	P51412	AT1G35680	R	0.32±0.04
21	50S ribosomal protein L24 (RPL24)	P92959	AT5G54600	R	0.26±0.02
22	50S ribosomal protein L31 (RPL31)	Q9FWS4	AT1G75350	R	0.44±0.04
23	Ribosomal L29 family protein (RPL29)	B9DH43	AT5G65220	R	0.24±0.03
	**Transportation (2)**				
24	Heavy metal transport/detoxification superfamily protein	Q93VK7	AT5G14910	S	0.52±0.04
25	Triose phosphate/phosphate translocator (TPT)	F4KG18	AT5G46110	IE	0.60±0.04
	**Unknown (1)**				
26	Uncharacterized protein	Q0WMN5	AT3G49140	Chl	0.68±0.04

^a^ Numberical list of dark-responsive proteins.

^b^ Protein name and the abbreviation commonly used for the protein. The proteins were classified according to their functions.

^c^ Protein number given by Uniprot_Arabidopsis database.

^d^ Gene number, by converting identified protein ID to gene number in TAIR database.

^e^ Subcellular localization of each chloroplastic protein according to PPDB database.

^f^ D/L Ratio ± SD is shown as protein ratios with standard deviations.

^g^ Functional group with number of proteins in this group. The abbreviations: D, dark-treated samples; L, control samples. SD, standard deviation. D/L means protein abundance value of dark sample divided by value of control sample. SD: standard deviation. Chl: chloroplast; IE: envelope-inner-integral; S: plastid stroma; R: plastid ribosome; N: plastid nucleoid.

### Characteristics of Proteins in Response to Darkness

We identified 15 darkness-responsive photosynthetic proteins in chloroplasts, including photosystem II-related proteins, photosystem I proteins, electron carriers, and one stress-related protein Rieske (2Fe-2S) domain-containing protein (PSB33) (Table 2). Among them, three chlorophyll *a*/*b* binding proteins, photosynthetic electron carriers (ferredoxin (Fd), two protein gradient regulation-5 (PGR5)-like proteins (PGRL1A and PGRL1B), and Photosystem I P700 chlorophyll a apoprotein A1 (PSAA) were increased under darkness. Besides, the thylakoid-associated kinases STN7 and STN8 were increased under darkness, which were essential for photosynthetic acclimation [39]. Moreover, PsbQ and three members of PsbP family (PsbP, PsbP-like protein 1 (PPL1) and PsbP domain-containing protein 5(PPD5)) were all increased under darkness. Among these proteins, PsbP and PsbQ play important roles in the stabilization of PSII [40–41]. PPL1 is required for efficient repair of photodamaged PSII and would function in the dynamic life cycle of PSII under the process of photoinhibition. PPD5 protein was suspected to function in the strigolactone biosynthetic pathway [42]. In addition, thylakoid curvature 1A protein (CURT1A) was increased under darkness, which would facilitate granum stacking and impose constraints on photosynthesis [43]. Interestingly, we also found three darkness-increased chlorophyll synthesis-related enzymes, including coproporphyrinogen-III oxidase 1 (CPOX), magnesium-protoporphyrin IX monomethyl ester cyclase (CRD1), and protochlorophyllide reductase like protein (POR C). One carotenoid synthesis-related enzyme (lycopene beta/epsilon cyclase protein (CrtL)) was increased under darkness.

We also found 15 darkness-increased proteins that were involved in carbohydrate metabolism and other metabolisms (Table 2). They were (1) seven Calvin cycle-related enzymes, i.e, RuBisCO large subunit (RBCL), RuBisCO small subunit (RBCS), and sedoheptulose-1,7-bisphosphatase (SBPase), transaldolase-like protein (TAL), TPI, fructose-bisphosphate aldolase (FBA), and fructose-bisphosphatealdolase 3 (FBA3); (2) three enzymes in chloroplastic lipid metabolism, pyruvate kinase 1(PKP1), phosphoenolpyruvate carboxylase family protein (PEPCK), and dihydrolipoyl dehydrogenase 1 (LPD1); (3) two amino acid (glutamine and arginine) metabolism-related enzymes, glutamine synthetase (GS) and carbamoyl-phosphate synthase large chain (Ven3); (4) isoprenoids metabolism-related protein, 4-hydroxy-3-methylbut-2-enyl diphosphate synthase (HDS); (5) a nitrogen metabolism-related enzyme, ferredoxin-nitrite reductase (NiR); and (6) Amidophosphoribosyltransferase 2 (ATase 2), which catalyzes the first step in purine nucleotide biosynthesis.

The darkness affected gene expression and protein synthesis in chloroplasts. Three RNA-binding proteins showed a decline in abundance, which were chloroplast RNA-binding protein 33 (PDE322), 31-kDa RNA binding protein (RBP31), and RNA-binding (RRM/RBD/RNP motifs) family protein (PSRP2). Besides, pTAC5 was reduced under darkness. pTAC5 is a component of plastid transcriptionally active chromosome proteins [44], which plays an important role in maintaining plastid-encoded RNA polymerase (PEP) function [45]. However, the increased CCR-like (CCL) protein was involved in post transcriptional processes, such as circadian mRNA oscillations in the Arabidopsis [46]. Moreover, 17 chloroplastic ribosome proteins and a translation initiation factor IF-2 (FUG1) in chloroplast translational apparatus were reduced (Table 3), but one ribosome-recycling factor (RRF) was increased under the dark treatment (Table 2). In addition, six darkness-increased proteins were involved in protein folding (i.e., peptidyl-prolylcis-trans isomerase FKBP19, FKBP16-1, FKBP16-4, and CYP20-3) and protein degradation (i.e., FtsH 1 and DEG 8).

The proteins involved in transport and signaling were altered in abundance under dark treatment. An outer envelope protein 64 (Toc64) and a thylakoid localized preprotein translocase subunit SCY1 were increased, but the abundances of three transporters were varied, including the increased inorganic phosphate transporter 2–1 (PHT2;1) and K^+^ efflux antiporter 3 (KEA3), as well as the decreased triose phosphate/phosphate translocator (TPT) and heavy metal transport/detoxification superfamily protein (Table 3). Importantly, five darkness signaling components were all increased, i.e, three GTP-binding protein of Ras superfamily (two rhodanese/cell cycle control phosphatase superfamily proteins and a rhodanese-like domain-containing protein 4 (TROL)), and two jasmonate acid (JA) synthesis-related proteins (allene oxide synthase (AOS) and allene oxide cyclase 4 (AOC4)). In addition, two darkness-induced proteins were involved in stress and defense, including resistance to phytophthora 1 protein (RPH1), acclimation of photosynthesis to environment protein (APE1) (Table 2).

### Evaluation of Protein Abundance by Western Blotting Analysis

**Fig 7:**
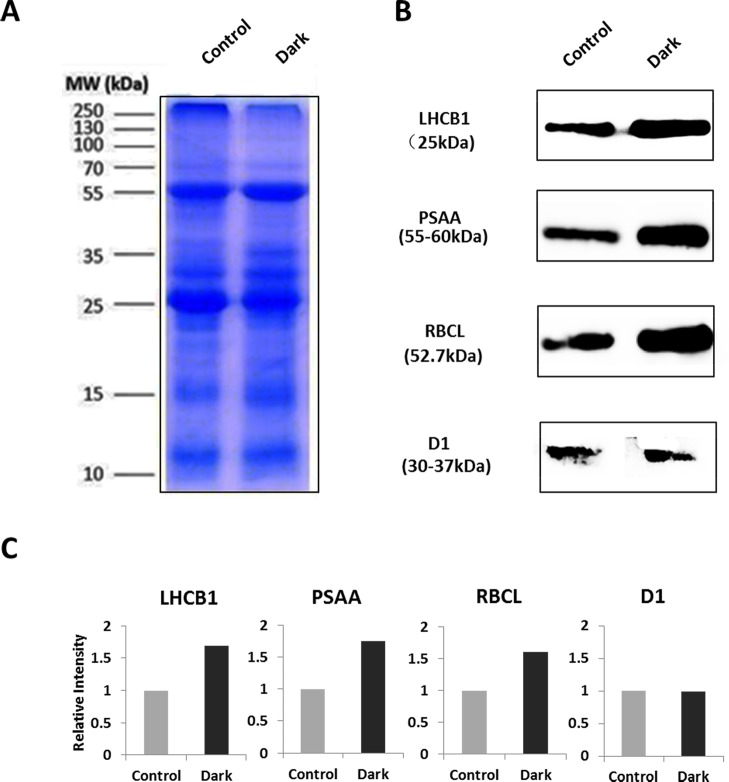
Western blotting analysis of selected darkness-responsive proteins in chloroplasts. (A) Coomassie stained gel image showing equal loading of protein samples (10μg each lane) from control and dark-treated plants (MW, molecular weight). (B) Representative Western blotting images of different proteins in chloroplasts (LHCB I, Light-harvesting chlorophyll protein complex II subunit B1; PSAA, Photosystem I P700 chlorophyll a apoprotein A1; RBCL, large subunit of Rubisco; and D1, photosystem II reaction center protein A). (C) Relative intensity of proteins shown in (B) determined from three Western experiments.

To evaluate the protein abundance, four representative proteins (LHCB1, PSAA, RBCL, and D1) were subjected to Western blotting analysis (Fig 7). Among them, LHCB1 (25 kDa), PSAA (55–60 kDa), and RBCL (52.7 kDa) were elevated, but the abundance of D1 didn’t show obvious changes under dark conditions (Fig 7). The protein abundance changes determined by Western blotting appeared consistent with that in TMT-based proteomic results (Table 2, S3 Table). This indicated that our proteomic results can reveal the protein abundance changes under dark treatment.

## Discussion

### Enhancement of Photosystem II Stability and Cyclic Electron Flow around PSI through Chlororespiration

**Fig 8:**
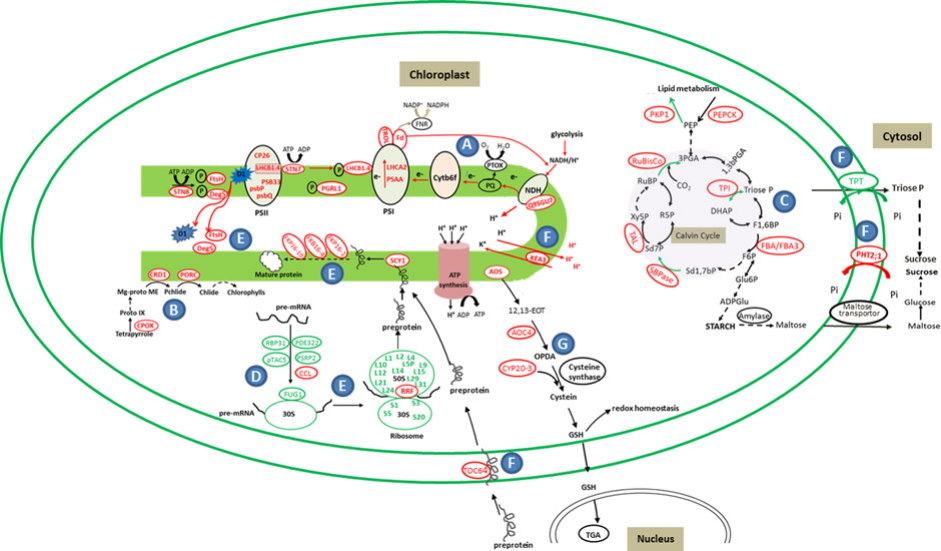
Schematic model of darkness-responsive proteins in chloroplasts of *Arabidopsis* leaves. The identified proteins were integrated into subcellular locations and functional pathways. Protein expression patterns were shown by marking the protein names in red (increased) or blue (decreased), and marking the reactions in red (increased) or blue (decreased) arrows. The solid line means one step. The dot line means multiple steps. A, Photosynthesis and chlororespiration; B, Chlorophyll synthesis; C, Calvin cycle; D, Transcription; E, Protein synthesis, folding and degradation; F, Fransportation; G, JA signaling; Abbreviations: ADPGlu, ADPglucose; AOC4, allene oxide cyclase 4; AOS, allene oxide synthase; CCL, CCR-like; Chlide, chlorophyllide; CP26, chlorophyll a-b binding protein; CPOX, Coproporphyrinogen-III oxidase 1; CRD1, Magnesium-protoporphyrin IX monomethyl ester [oxidative] cyclase; CYP20-3, cyclophilin 20–3; Cytb6f, cytochrome b6/f; DEG8, Protease DEG 8; DHAP, dihydroxyacetone phosphate; 12,13-EOT, 12,13-epoxy-octadecatrienoic acid; F6P, fructose-6-phosphate; F1,6BP, fructose-1,6-bisphosphate; FBA, fructose-bisphosphate aldolase; FBA3, probable fructose-bisphosphate aldolase 3; Fd, sulfite reductase [ferredoxin]; FKBP16-1, peptidyl-prolylcis-trans isomerase FKBP16-1; FKBP16-4, peptidyl-prolylcis-trans isomerase FKBP16-4; FKBP19, peptidyl-prolylcis-trans isomerase FKBP19; FNR, Fd:NADP^+^ oxidoreductase; FtsH, FtsH extracellular protease family protein; FUG1 Translation initiation factor IF-2; Glu6P, Glucose-6-phosphate; GSH, glutathione; KEA3, K+ efflux antiporter 3; LHCA2, Photosystem I light harvesting complex gene2 protein; LHCB1, light-harvesting chlorophyll protein complex II subunit B1; Mg-Proto, Mg-protoporphyrin; OPDA, (+)-12-oxophytodienoic acid; Pchlide, protochlorophyllide; PDE322, chloroplast RNA-binding protein 33; PEP, phosphoenolpyruvic acid; PEPCK, phosphoenolpyruvate carboxylase family protein; 1,3PGA, 1,3-bisphospoglycerate; 3PGA, 3-phosphoglycerate; PGRL1, PGR5-like protein 1; PHT2;1, inorganic phosphate transporter 2–1; PKP1, Plastidial pyruvate kinase 1; POR, Protochlorophyllide reductase like protein; PQ, plastoquinone pool; Proto-IX, protoporphyrin-IX; PSAA, photosystem I P700 chlorophyll a apoprotein A1; PSB33, Rieske (2Fe-2S) domain-containing protein; PsbP, photosystem II subunit P; PsbQ, photosystem II subunit Q; PSRP2, RNA-binding (RRM/RBD/RNP motifs) family protein; pTAC5, plastid transcriptionally active 5; PTOX, plastid terminal oxidase; R5P, ribulose-5-phosphate; RBP31, 31-kDa RNA binding protein; RRF, ribosome-recycling factor; RuBisCO, ribulose bisphosphate carboxylase; RuBP, Ribulose-1,5-bisphosphate; SBPase, sedoheptulose-1,7-bisphosphatase; SCY1, preprotein translocase subunit SCY1; Sd7P, sedoheptulose-7-phosphate; Sd1,7bP, sedoheptulose-1,7-bisphosphate; STN7, serine/threonine-protein kinase STN7; STN8, serine/threonine-protein kinase STN8; TAL, transaldolase-like protein; TGA, transcription factors; Toc64, Outer envelope protein 64; TPI, triosephosphate isomerase; TPT, triose phosphate/phosphate translocator; Triose P, Glyceraldehyde 3-phosphate; TROL, thylakoid rhodanese-like protein; TRX, thioredoxins; Xy5P, xylulose-5-phosphate.

The photosynthetic apparatus function as light energy collector and converter. Photosynthesis system reconfigures its components in response to environmental changes and metabolic needs [47]. Under dark treatment, the stability and repair of PSII is crucial for photosynthetic apparatus. Our proteomic results revealed that several dark-increased proteins were facilitated to stabilize and/ repair PSII complexes (Table 2; Fig 8). Among these proteins, STN7 can mediate LHCII proteins phosphorylation for regulating energy distribution between PSI and PSII [48, 49]. Besides, STN8 kinase can phosphorylate PSII core proteins D1, which functions as a signal for the degradation of photodamaged PSII core complex and replacement of phosphorylated D1 protein [50]. In addition, Deg and FtsH were cooperatively involved in the degradation of photodamaged and phosphorylated D1, being considered as a crucial protective mechanism to prevent the accumulation of photodamaged D1 proteins of PSII [51].

Besides, four increased PSII peripheral protein components (PsbP, PsbQ, PPL1, and PPD5) and one integral membrane protein PSB33 would either enhance the assembly or repair of PSII to prevent the production of reactive oxygen species and the consequent damage of the photosynthetic machinery under dark (Fig 8) [52–53]. In addition, chloroplasts of land plants contain grana comprised of approximately five to twenty layers of thylakoid membrane [54]. PSII is mainly located in the stacked grana thylakoids [55]. CURT1A can change membrane curvature in grana resulting in a contracted grana lumen [43], which would limit the movement of oxygen evolving complex (OEC) in order to stabilize PSII in dark [56]. Thus, the increased CURT1A was supposed to facilitate the stability and repair of PSII under dark treatment.

Chlororespiration allows for the production of ATP in the dark [11]. This pathway includes a plastid NDH complex (NDH), a plastid terminal oxidase (PTOX), and CEF around PSI [57]. In our results, the increased PSAA, Fd, PGRL1 (PGRL1A and PGRL1B) and STN8, implied that chlororespiration were enhanced under the dark treatment (Fig 8A). Fd and PSAA are important members for electron flow in PSI. It is well known that CEF and LEF share a number of common carriers (from plastoquinone to Fd), leading to be in competition with one another. The fraction of PSI involved in CEF in dark-adapted leaves can be close to 100%, but only 50% of PSI fraction participates in CEF in the light [58]. Thus, in the dark-treated leaves, CEF around PSI appeared a maximum when PS II is inhibited [58]. The main function of CEF is to increase the concentration of ATP, which is critic for the rate of the Calvin cycle after a period of dark adaptation [58]. The partition between LEF and CEF proposed to be essentially dependent on Fd as an electron carrier [59], and overexpression of Fd would induce CEF [60]. The PGRL1 can be phosphorylated by STN8 and the phosphorylated form of PGRL1 was supposed to be involved in the stabilization of CEF around PSI [57, 61]. In addition, the aforementioned STN7 can phosphorylate LHCII, resulting in translocation of LHCII to PSI. This led to the preferential increase of electron flow around PSI [62]. It has been reported that Arabidopsis *STN7* overexpression plants showed enhanced electron flow around PSI and higher performance of PSI than wild type in the dark [63]. Thus, the increased amount of PSAA, Fd, PGRL1 (PGRL1A and PGRL1B), STN8 and STN7 were in accordance with the enhanced CEF and reduced LEF revealed from higher value of Y(CEF)/Y(II) and lower efficiency of PSII in the dark (Table 1). In addition, chlororespiration requires the essential NDH complex to transfer reduced power [64]. A two-fold increased uncharacterized protein (Q9SGU7) in our results were predicted to be correlated with the subunit of NDH complex (http://atted.jp/data/), implying the growing evidence of chlororespiration.

### Conversion of Chlorophyll *a* to Chlorophyll *b* is Promoted under Darkness

Chlorophyll *a* and chlorophyll *b* as light-absorbing pigments enable efficient harvesting of light energy [65]. As a component of the photosynthetic machinery, chlorophylls occur in the pigment-protein complexes localized in light-harvesting antennas and photosynthetic reaction centers of PSI and PSII [65]. The absence of Chls would lead to the rapid degradation of chlorophyll-binding proteins [66]. The chlorophyll *a* and chlorophyll *b* have different roles in chloroplasts. Chlorophyll *a* can bind to the core antenna complexes to carry out photosynthetic charge separation [67], and the major role of chlorophyll *b* is to stabilize the peripheral antenna complexes [68]. It was found that accumulation of LHC was increased in Arabidopsis plants in which chlorophyll *b* biosynthesis was genetically enhanced [69]. However, it was reduced in chlorophyll *b* deficient mutant plants [70]. Moreover, it has been observed that chlorophyll *a* was converted to chlorophyll *b* in angiosperms under darkness [71], which would result in the reduced accumulation of PSII core complexes [72]. In our results, we found total chlorophyll content was stable, but chlorophyll *a*/*b* and Fv/Fm were reduced (Table 1, Fig 2). This implied that parts of chlorophyll *a* was converted to chlorophyll *b*, leading to the reduced capability of PSII core complexes under dark treatment, while the increased chlorophyll *b* would enhance the stability of the peripheral antenna complexes. It was reported that Chlorophyllide a oxygenase (CAO) was the only known enzyme responsible for the formation of Chlorophyll(ide) *b* [73]. However, we didn’t detect the abundance change of CAO in dark (S1 Table). It was found that CAO activity was mainly regulated by protein stability but not protein abundance [74]. When full-length CAO cDNA was overexpressed in Arabidopsis, the conversion of chlorophyll *a* to *b* was only slightly changed [75–76]. This implied that CAO activity was regulated under post-transcriptionally and/or post-translational modification. Therefore, the conversion mechanism of Chl *a* to Chl *b* regulated by CAO needs to be further investigated.

Importantly, we found several chlorophyll biosynthesis-related enzymes (CPOX, CRD1, and PORC) were increased under dark treatment. CPOX catalyzes the oxidative decarboxylation of coproporphyrinogen III to form protoporphyrinogen IX chlorophyll biosynthesis, which is shared in heme biosynthesis, while CRD1 and PORC catalyze the formation of protochlorophyllide (Pchlide) and chlorophyllide (Chlide) in chlorophyll synthesis, respectively (Fig 8B). PORC is predominantly present as one isoform of POR in fully matured green tissues [77]. The activity of PORC was light dependent [78], which is different with CPOX and CRD1. Under darkness, chlorophyll biosynthesis halts at the step of Pchlide, the immediate precursor of Chlide, which is catalyzed by PORC. Arabidopsis is unable to synthesize chlorophyll in the dark [12]. PORC binding with Pchlide forms a ternary Pchlide-POR complex to avoid the overproduction of ^1^O_2_ under darkness [79]. The increases of these enzymes would be the enhancement of chlorophyll biosynthesis potential under darkness, which is consistent with the phenomenon of increased abundance of various enzymes for chlorophyll biosynthetic pathway in Arabidopsis PORC overexpression lines in the dark [80].

### Carbohydrate Metabolism and Transport under Darkness

Chloroplast is the important organelle for carbon fixation and carbohydrate metabolism. Carbon is assimilated by Calvin cycle in the day and the starch is mainly degraded at night, because the activities of most enzymes for carbon fixation are light dependent, such as RuBisCO [81] and SBPase [82]. We found the abundances of several enzymes in Calvin cycle were increased, but the activities of two key enzymes (i.e. RuBisCO and TPI) were reduced under the dark treatment (Fig 8C). It is known that the protein abundance sometimes does not correlate well with its enzyme activity, because the enzyme activity is not only decided by its abundance, but also its structure, conformation, and post-translational modification. The activation of RuBisCO *in vivo* requires the presence of RuBisCO activase in the light, while the activity of RuBisCO activase is light/dark regulated and the most dramatic at physiological ratios of ADP/ATP (≅0.33) typical of values were observed in the light [83]. In addition, it was also reported that the catalytic potential of RuBisCO in the dark correlated with the preceding enzyme phosphoribulokinase (PRK). PRK remained inactive in the dark and did not provide any RuBP substrate [84]. This would result in the reduced activity of RuBisCO in dark. All these implied that several enzymes in Calvin cycle were accumulated, but their activities were inhibited at night (Fig 3C and 3D). This is consistent with the notion that the Calvin cycle is regulated mainly by the alteration of enzyme activity, but not protein abundance [85–86].

It is well known that the starch breakdown in amylolytic way mainly takes place at night, due to the amylase activity with obvious diurnal oscillation [87]. In our results, the increased sugar content was mainly due to the active starch breakdown at night (Fig 3A and 3B). In addition, relatively low pH is optimal for amylases. Thylakoid KEA3 can mediate K^+^/H^+^ exchange, resulting in H^+^ flow into stroma and K^+^ uptake into the thylakoid lumen [88]. We found an increased KEA3 under the dark treatment, which was involved in keeping low pH in stroma for starch breakdown. In addition, we also found the induced CEF activity (Table 1), which would make acidification of the lumen, leading to elevated H^+^ to be exported to the stroma by KEA3 in darkness.

Besides, we found a darkness-decreased TPT, which is the transporter for triose phosphate export from chloroplast to the cytosol [89]. It was reported that, in antisense TPT plants, carbon exportation from chloroplast was reduced in the day, but enhanced at night [90]. At night, active hexoses (e.g., glucose and maltose) exportation to cytosol is mainly depended on glucose transporter [91]. In addition, the increased PHT2;1 under dark treatment would play a role as an alternative mechanism for phosphate import into chloroplast stoma [92]. The high concentration of phosphate (Pi) in the stroma may promote starch degradation reaction by inhibiting ADP-glucose pyrophosphorylase (a key enzyme of starch synthesis) [93].

### Chloroplast Gene Expression, Protein Synthesis and Processing under Darkness

The plastid-encoded RNA polymerase (PEP) complex is the major RNA polymerase in mature chloroplasts, which is composed of four core subunits (rpoA, rpoB, rpoC1, and rpoC2) and additional protein factors (sigma factors and polymerase associated protein(PAPs)) encoded in the nuclear genome. The majority of PAPs [94] and sigma factor genes of Arabidopsis were light induced [95]. Plastome-wide PEP-DNA association was reduced during the dark period [96]. Therefore, light plays important roles in the regulation of plastid gene transcription.

All four plastid-encoded RNA polymerase (PEP) core subunits (Rpo-A, B, C1, and C2) were constitutively expressed in chloroplasts, and remained unchanged under the dark treatment (S1 Table). Some nuclear-encoded proteins (i.e., PDE322, RBP31, PSRP2 and pTAC5) involved in post-transcriptional process were reduced under dark treatment (Table 3, Fig 8D).

We found that 17 chloroplastic ribosome proteins were decreased under darkness (Table 3, Fig 8E), implying that the chloroplast translation machinery was strongly repressed. This is consistent with that protein synthesis in plastid is especially light dependent [97–98], due to plastidic ATP-ADP ratio is low in the dark as compared with in the light [13]. However, the increased ribosome-recycling factor (RRF) under dark treatment can increase the synthesis rate of the chloroplast-encoded proteins, because RRF can facilitate ribosome recycling from the last round of translation into the the next round of translation [99].

In addition, the four darkness-increased proteins in our results contain peptidyl-prolyl cis-trans isomerase (PPIase) domain, which are thought to be essential for protein folding during protein synthesis [100] (Fig 8E). Among them, the increases of FKBP16-1 could enhance the acclimation of photosystem. It was reported that overexpression of AtFKBP16-1 showed increased stability of PSI-LHCI and PSI-LHCI-LHCII due to the regulation of PsaL stability [101], which was advantageous for chlororespiration around PSI.

The majority of chloroplastic proteins are synthesized in cytoplasm, and then transported to chloroplast. We found two increased proteins (Toc64 and SCY1) in the dark treatment, which were involved in protein transport (Fig 8F). Toc64 is a docking protein in chloroplast envelope, which functions in nuclear encoded preprotein import from cytosol into chloroplasts [102]. SCY1 is localized to the thylakoids, in charge of preprotein localization to thylakoid [103]. Maize scy1 mutant showed impairment in thylakoid biogenesis [104]. Mutants lacking components of the chloroplastic protein import apparatus showed decreased nuclear-encoded chloroplastic proteins [105]. In our results, increased Toc64 and SCY1 under the dark treatment would facilitate the import of increased nuclear-encoded proteins into the chloroplasts.

### Redox and JA Signaling under Darkness

Redox homeostasis is crucial for functional coupling of light and dark responses in chloroplast, modulating numerous biochemical processes through changes in protein activities [106–108]. The change in the redox state of thiol groups in proteins is an important pathway underlying redox regulation [109]. Rhodaneses catalyze the transfer of a sulfane sulfur atom from thiosulfate to cyanide *in vitro* [110], which regulates redox changes of thiol groups to maintain the cellular redox homeostasis [111]. Furthermore, several rhodanese-like proteins have been identified. The thylakoid rhodanese-like protein (TROL) has a docking site for Fd: NADP^+^ oxidoreductase (FNR) to facilitate the reduction of chloroplast Trx [112] in the light but not dark, then Trx-dependent thiol modulation ensures redox control on the expression of genes encoding chloroplast proteins. Besides thylakoid localization, TROL is localized at the inner envelope of chloroplast, and required for regulation of nuclear-encoded protein import across the inner envelope [113]. In our results, two rhodaneses and one rhodaneses-like protein (TROL) were increased in chloroplast under dark treatment, indicating the nuclear-encoded protein expression and transport into chloroplasts were enhanced under dark treatment.

The redox homeostasis in chloroplasts is regulated by JA signaling [114]. We found two enzymes (AOS and AOC4) involved in JA synthesis [115] and a receptor of JA (CYP20-3) were induced in chloroplasts under dark treatment. This would result in increases of thiol metabolites and enhance redox capacity in chloroplast in order to coordinate the expression of a subset of genes (Fig 8G). However, it has been reported that plant sensitivity to JA was lower in the night than in the day [116]. It is important for plant to save energy in order to maintain growth in the night [117].

## Conclusion

Chloroplasts have evolved a fine-tuned mechanism in response to the light and dark cycle. In this study, the quantitative proteomics results together with corresponding physiological characteristics revealed that multiple pathways were involved in the darkness response. They include: (1) electron flow through chlororespiration mainly accounted for ATP production in darkness; (2) PHT2;1 for sugar transport was induced, but triose-phosphate translocator was reduced under darkness; (3) carbon fixation was inactive under darkness; (4) protein import and sorting into chloroplast were active under darkness; and (5) redox and JA signaling pathways were active for metabolic homeostasis and gene expression regulation. The results have provided invaluable information towards understanding chloroplast functions in the light and dark cycle.

## Supporting Information

S1 Fig Quality control of the mass spectrometry data. (A) Mass error distribution of the identified peptides; (B) Peptides length distribution.
(DOCX)

S1 Table The identified chloroplastic proteins in PPDB database and/or AT-CHLORO database and their quantitative information (XLSX)

S2 Table The identified proteins that are not found in PPDB database and AT-CHLORO database and their quantitative information. (XLSX)

S3 Table Darkness-increased proteins in chloroplasts identified in this study. (XLSX)

S4 Table Darkness-decreased proteins in chloroplasts identified in this study. (XLSX)
