# Supplementary material for: Proteomic Insight into the Response of Arabidopsis Chloroplasts to Darkness
Source: PLoS One. 2016 May 3;11(5):e0154235. doi: 10.1371/journal.pone.0154235 (PMC4854468; doi:10.1371/journal.pone.0154235)
Supplement: S1 Fig — (A) Mass error distribution of the identified peptides; (B) Peptides length distribution. (DOCX) [file pone.0154235.s001.docx]

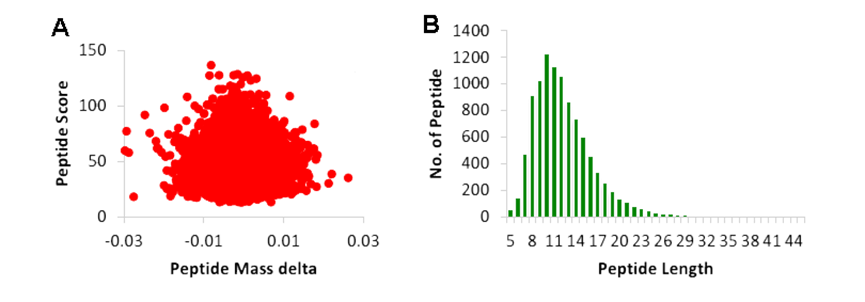


**S1 Figure. Quality control of the mass spectrometry data.** (A) Mass error distribution of the identified peptides; (B) Peptides length distribution.
